# Acetylation of lysine 182 inhibits the ability of *Mycobacterium tuberculosis* DosR to bind DNA and regulate gene expression during hypoxia

**DOI:** 10.1038/s41426-018-0112-3

**Published:** 2018-06-13

**Authors:** Jing Bi, Zongchao Gou, Fengzhu Zhou, Yiqing Chen, Jianhua Gan, Jun Liu, Honghai Wang, Xuelian Zhang

**Affiliations:** 1 State Key Laboratory of Genetic Engineering, School of Life Science, Fudan University, Shanghai, 200438 China; 2 Department of Molecular Genetics, Faculty of Medicine, University of Toronto, Toronto, ON M5G 1M1 Canada

## Abstract

The DosR regulon is believed to be a key factor in latency adaptation of *Mycobacterium tuberculosis* and is strongly induced by multiple stresses, including hypoxia. Previous studies have revealed reversible acetylation of the conserved core DNA-binding lysine residue 182 (K182) of DosR in *M. tuberculosis*. In this study, we demonstrated that acetylated K182 plays an important role in the DNA-binding ability of DosR and that acetylation of K182 completely abolished the affinity of DosR for DNA in vitro. Antibodies that specifically recognized acetyllysine at position 182 of DosR were used to monitor DosR acetylation. We found that in vitro acetylation of K182 could be removed by deacetylase Rv1151c and that either the deacetylase*-*deletion strain ∆*npdA* or treatment with a deacetylase inhibitor resulted in increased levels of K182 acetylation in vivo. The physiological significance of DosR acetylation was demonstrated by decreased levels of acetylated K182 in *M. tuberculosis* in response to hypoxia and by the effects of K182 acetylation on the transcript levels of DosR regulon genes. Since the DosR regulon plays a critical role during host infection by *M. tuberculosis*, our findings suggest that targeting DosR acetylation may be a viable strategy for antituberculosis drug development.

## Introduction

Tuberculosis (TB) is a potentially deadly bacterial infectious disease that worldwide caused 1.8 million deaths and 10.4 million new infections in 2016^1^. As a successful bacterial pathogen, *Mycobacterium tuberculosis* (*M. tuberculosis*) can adapt to the host environment and transition into a dormant or latent infection^2,3^. Latent infections represent major disease reservoirs and are a source of potential risk^4^. The DosR regulon is believed to be a key factor in the latency adaptation of *M. tuberculosis*^4–6^. DosS/DosR, also called DevS/DevR, is a two-component regulatory protein complex and is strongly induced by multiple stresses such as hypoxia, carbon monoxide, and nitric oxide^7–11^. In the granulomas produced in the host body, DosT and DosS are sensor kinases that detect environmental stresses such as hypoxia^12–14^. As a transcription factor, DosR receives signals from DosT/S and then initiates a latent infection^8,15–17^. There are approximately 48 genes induced by DosR that assist in the development of latency in *M. tuberculosis*^8,16^. Therefore, DosR regulon function is believed to be essential for bacterial survival during latency.

The mechanism of DNA binding by DosR to regulate the transcription of dormancy-related genes and the adaptation to latency by *M. tuberculosis* have been investigated over the past few years. A conserved histidine of DosT/S becomes phosphorylated when *M. tuberculosis* experiences conditions of hypoxia, and the phosphate group is then transferred to Asp54 of DosR^18,19^. Dimerization is an essential step for the activation of DosR, and phosphorylation advances the dimerization process^19,20^. However, unphosphorylated DosR can interact with DNA at high concentrations^14,21^. The phosphorylation-defective DosR D54E protein functions similarly to phosphorylated DosR, which enhances DNA-binding affinity in vitro^14^. Previous studies have suggested that, in addition to phosphorylation modification, there may be other unknown mechanisms of DosR regulation. Additionally, researchers have shown that there are no latency-relevant phenotypes or physiological changes when DosR is overexpressed in *M. tuberculosis* strains H37Rv ΔdosR or H37Rv^22,23^. These observed phenomena lead to questions regarding the mechanism of DosR activation and emphasize the need for additional studies aimed at explaining the undefined mechanisms.

Protein acetylation plays an important role in bacterial chemotaxis, metabolism, DNA replication, virulence, and other cellular processes in *Escherichia coli*, *Salmonella enterica*, *Saccharopolyspora erythraea*, and *Salmonella* sp.^24–29^. We have also discovered that lysine acetylation plays a critical role in the regulation of central carbon metabolism in *M. tuberculosis*^30^, suggesting that protein acetylation is a universal posttranslational modification for regulating protein functions in prokaryotes. Acetylation of lysine residue 182 (K182) of DosR was detected in both our acetylome data^30^ and by Liu and colleagues^31^. The crystal structures show that K182 is one of three core DNA-binding amino acids (K182, N183, and K179)^20^. Therefore, we hypothesized that DNA-binding affinity may also be regulated by the acetylation of K182 in DosR and that it influences the transcription of downstream proteins. In this study, we demonstrated that acetylation of residue K182 abolished the DNA-binding ability of DosR and further altered the transcription of DosR-regulated genes.

## Results

### DosR K182Q mutation nullified protein–DNA binding ability

**Fig. 1:**
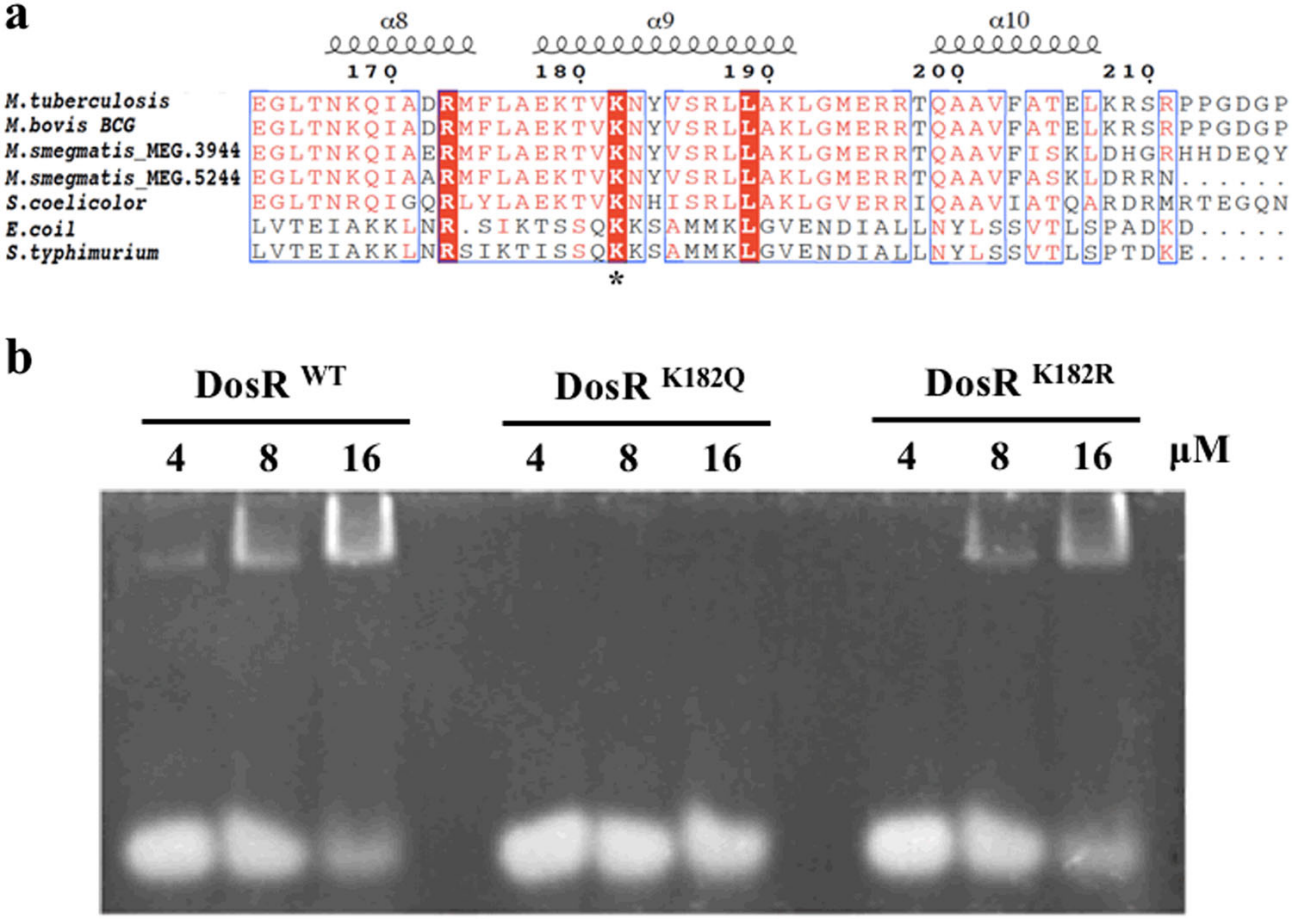
K182Q mutant abolishes the DNA-binding ability of DosR. **a** Conservation analysis of the DosR sequence surrounding K182 from various bacteria, including *Mycobacterium*, *Streptomyces*, *Escherichia*, and *Salmonella* strains. Asterisk indicates the conserved lysine residues. **b** Electrophoretic mobility shift assay (EMSA) analysis using DosR and derivatives and a consensus DosR regulon 20-bp DNA fragment^8^. EMSA was used to evaluate the DNA-binding abilities of DosR and its derivatives at three concentrations (4 μM, 8 μM, and 16 μM). EMSA results are representative of three independent experiments

The dormancy survival regulon DosR is believed to play a key role in latency adaptation of *M. tuberculosis* in response to hypoxic conditions when under attack by the host immune system^6,11^. Our previous studies on the lysine acetylome indicated that the differential pattern of protein acetylation of *M. tuberculosis* between aerobic and hypoxic cultures is reversible for DosR K182^30^. It has been reported that K182 is an indispensable residue for DNA binding by DosR^32,33^. Sequence alignment showed that K182 is highly conserved in bacteria (Fig. 1a). Hence, we hypothesized that acetylation of K182 may change the DNA-binding affinity and influence the transcription of downstream proteins.

To determine whether acetylation of K182 affected the function of DosR, we generated site-specific mutants in which K182 was substituted with arginine (DosR^K182R^) to mimic unacetylated lysine or with glutamine (DosR^K182Q^) to mimic acetyllysine. Electrophoretic mobility shift assays (EMSA) were performed by incubating a 20-bp DNA fragment with a consensus sequence for the DosR regulon^20^, along with purified wild-type DosR (DosR^wt^) or DosR mutants, in order to analyze the different DNA-binding affinities of the DosR derivatives. The EMSA analysis showed that with increasing DosR concentrations, the amount of bound DNA increased. DosR^K182R^, in which the arginine substitution mimics unacetylated lysine but maintains a positive charge, had a DNA-binding capacity similar to that of DosR^wt^ (Fig. 1b). In contrast, the DosR^K182Q^ mutation, in which the glutamine substitution mimics the acetylated form through neutralization of the positive charge, showed a reduced DNA-binding affinity compared with that of DosR^wt^ (Fig. 1b). Therefore, the abolition of the DNA-binding ability of the K182Q mutant suggested that acetylation of K182 may be associated with its ability to bind DNA.

### Site-specific acetylation of K182 abolished DosR-DNA binding

**Fig. 2:**
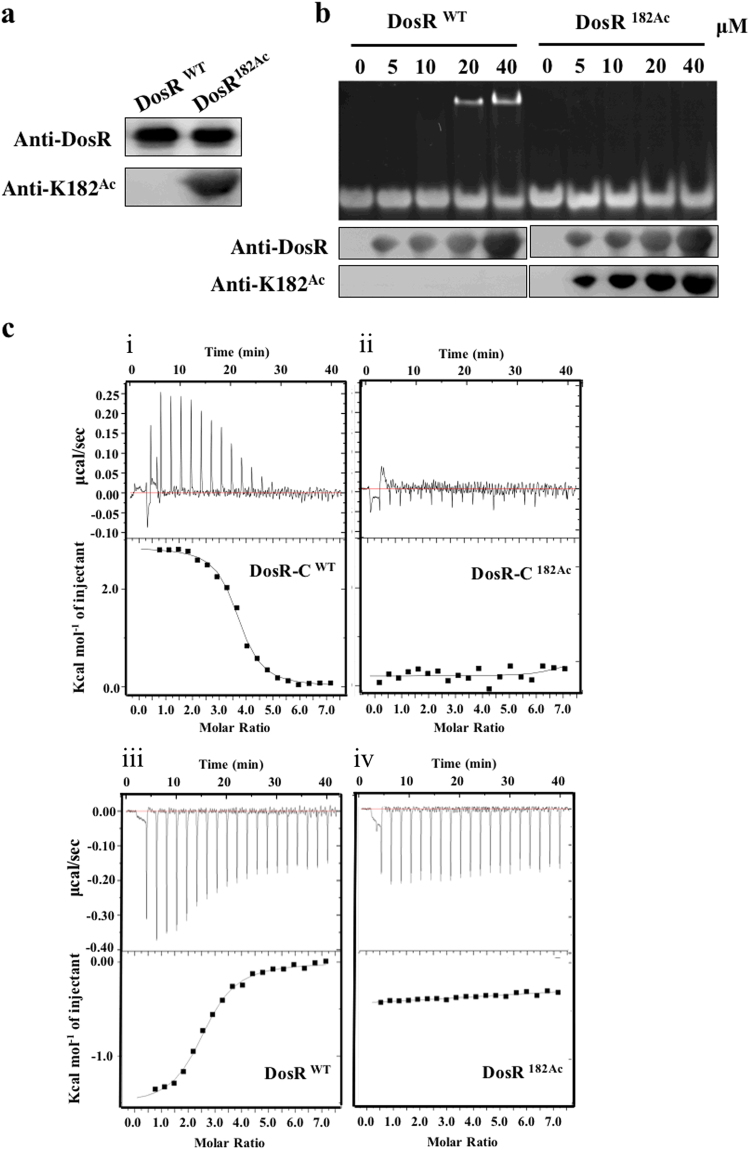
Acetylation of K182 abolishes the binding of DosR to its regulon promoter. **a** Western blot analysis of purified wild-type DosR (DosR^wt^) or DosR protein in which K182 was acetylated (DosR^182Ac^). Antibodies specific for DosR (anti-DosR, 1:10,000) and acetylated K182 peptides (anti-K182^Ac^, 1:1000) were used, and the exposure time was 30 s (for anti-K182^Ac^) or 3 s (for anti-DosR). **b** DNA-binding abilities of DosR and DosR^K182Ac^. Electrophoretic mobility shift assay (EMSA) was used to evaluate the DNA-binding abilities of DosR and its derivatives at the indicated concentrations (lanes 2–5 and lanes 7–10) to a 20-bp DNA fragment. The acetylation level of DosR^K182Ac^ in EMSA was tested using western blot. **c** Binding of full-length DosR or DosR^K182Ac^ and the C-terminal domain of DosR (DosR-C, residues 144–217) or DosR-C^K182Ac^ to DNA by isothermal titration calorimetry (ITC). The representative raw ITC data and the fitted binding curves are shown for the proteins DosR (i), DosR^K182Ac^ (ii), DosR-C (iii), and DosR-C^K182Ac^ (iv). Results are representative of at least two independent experiments

As an important type of posttranscriptional modification, Nε-acetylation is reversible and dynamic and allows precise modulation of protein function^34^. To further determine whether the acetylation of K182 had an important role in DNA binding of DosR, a system for genetically encoding N_Ɛ_-acetyllysine was used as previously described^35^. In addition, we prepared a specific polyclonal antibody against DosR containing acetylated K182 (DosR^182Ac^). This antibody demonstrated excellent specificity; it did not react with DosR^wt^ protein but did react with DosR^182Ac^. This antibody was used to confirm the successful incorporation of acetylated K182 by western blot assay (Fig. 2a). Subsequently, EMSA was used to assess the differential DNA-binding affinity between DosR^wt^ and DosR^182Ac^. As shown in Fig. 2b, DosR^wt^ without site-specific acetylation bound more DNA as concentrations were increased, consistent with DosR^182R^ (Fig. 1b). The site-specific acetylation protein DosR^182Ac^, however, completely lost its DNA-binding ability (Fig. 2b). To confirm the EMSA results, isothermal titration calorimetry (ITC) was used to examine the DNA binding of DosR^wt^ and DosR^182Ac^. Since the amino acid residues 147–217 of the C-terminal domain fragment serve as the functional unit for DNA binding^20^, in addition to full-length DosR and DosR^182Ac^, we also purified amino acid residues 147–217 from the C-terminal domain fragments of DosR (DosR-C) and DosR-C^182Ac^. The ITC analysis showed that both DosR^wt^ and DosR-C had very high DNA-binding affinities, with *Kd* values of 3.17 ± 0.29 µM and 0.52 ± 0.04 µM, respectively (Fig. 2c–i and 2c–iii). Though further verification is required, we believe that the N- and C-terminal domains of DosR^wt^ may participate in certain interactions that inhibit the DNA binding of the C-terminal and result in the slightly higher *Kd* value of full-length DosR^wt^. These results indicated that the K182 residue plays a crucial role in DNA binding, and this finding was consistent with that of the previous structural study^20^. Notably, K182 site-specific acetylation of both DosR and DosR-C completely disrupted their DNA-binding abilities (Fig. 2c–ii and 2c–iv), indicating that acetylation of DosR K182 abolished the DNA-binding ability of DosR.

**Fig. 3:**
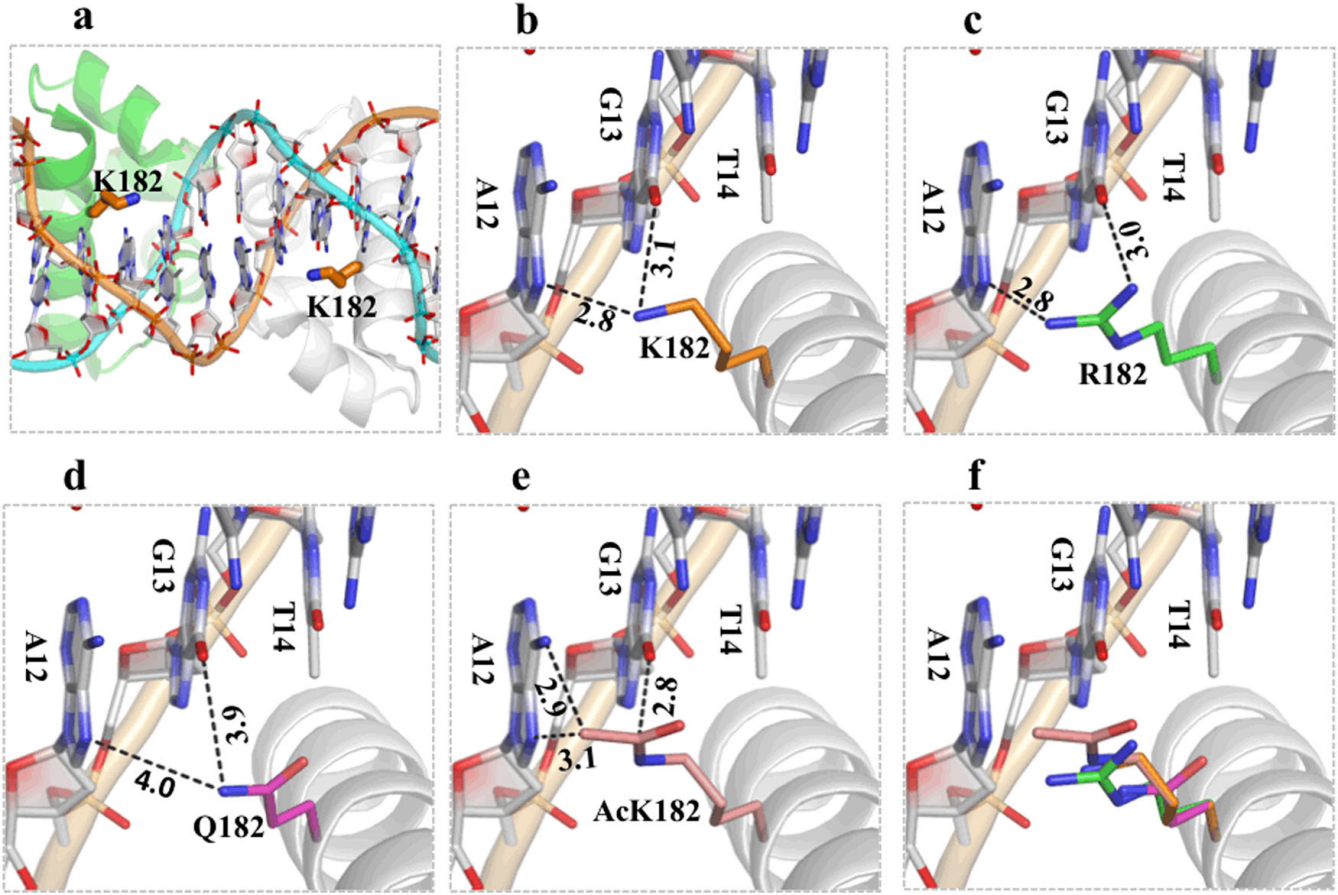
Molecular modeling of K182 acetylation in DosR. The structure of DosR was modeled using the crystal structure (PDB_ID: 1ZLK) of *M. tuberculosis* DosR-C in complex with DNA as its template. **a** The data show how K182 is located in the middle of helix 9, and its side chain points toward the nucleotide bases of DNA from the major-groove side. **b** Two H-bond interactions between K182 and A12 or K182 and G13 play the most important role in DNA recognition. **c** The K182R mutant can form two H-bonds with the target DNA to maintain its DNA-binding ability. **d** The K182Q mutant increases the distance between K182 and A12 or K182 and G13 and weakens the interaction with the target DNA. **e** The acetylated K182 has close contact between the side chain of the acetylated K and the DNA nucleotide bases. The superimposition shows the change in distance between the side chains at position 182 for different residues and the target DNA (**f**)

As revealed from the crystal structure (PDB_ID: 1ZLK) of the *M. tuberculosis* DosR-C complex with DNA, DosR-C functions as a dimer^19,20^. The DosR-C dimer forms several H-bond interactions with the DNA backbone phosphate groups but forms limited interactions with DNA nucleotides. K182 is located in the middle of helix 9, and its side chain is pointed toward the DNA bases from the major-groove side (Fig. 3a). The Nz atom of each K182 residue forms two H-bond interactions (one with the N7 atom of A12, and the other with the O6 atom of G13) and plays the most important role in target-sequence recognition (Fig. 3b). The basis for the in vitro K182 mutation or acetylation effects was revealed by structure-based modeling (Figs. 3c–3f). Similar to K182, R182 (for DosR^K182R^ mutant) formed two H-bonds with the target DNA; although the details of the interactions were not identical, these H-bonds helped maintain the DNA-binding ability of DosR^K182R^ (Fig. 3c). Compared with that of Lys, the side chain of Gln is shorter. Replacing K182 with Q182 (DosR^K182Q^ mutant) significantly increased the distances and weakened the interaction with the target DNA (Fig. 3d). While their sizes are comparable, the acetylated Lys residues were much more hydrophobic than the Arg residues, possibly because of the extremely close contact between the acetylated-K side chain and the DNA nucleotide bases. The result was that the DNA was “pushed” away (Fig. 3e).

### DosR^182Ac^ could be deacetylated by nicotinamide adenine dinucleotide (NAD^+^)-dependent deacetylase

**Fig. 4:**
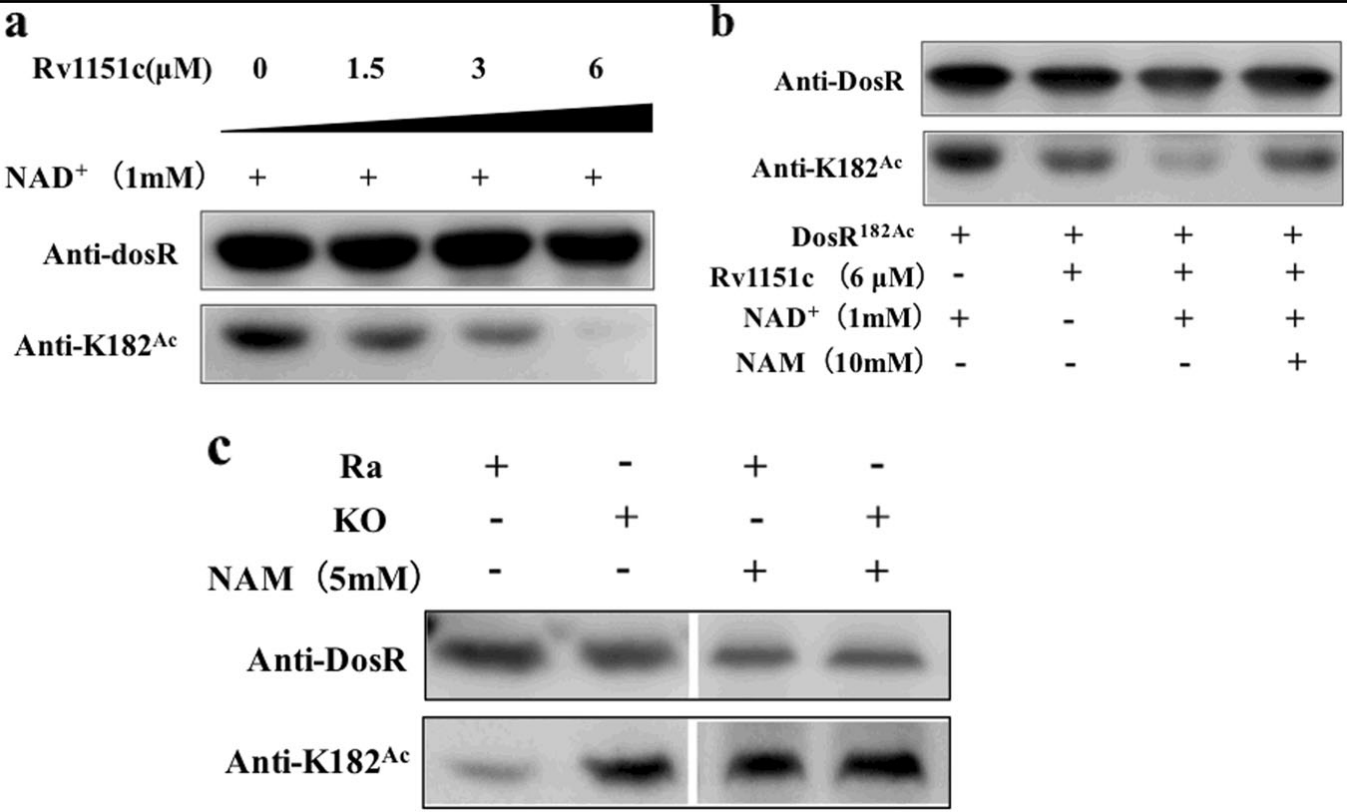
DosR can be deacetylated in vitro and in vivo. **a** Rv1151c deacetylates DosR in a dose-dependent manner in vitro. Purified DosR^182Ac^ (8 µM) was incubated with NAD^+^ (1 mM) and Rv1151c at the indicated concentrations (1.5 µM, 3 µM, and 6 µM) at 25 °C for 2 h. The acetylation levels were evaluated by western blot with anti-DosR (1:10,000) at 3-s exposure and anti-K182^Ac^ (1:1000) at 30-s exposure. The results are representative of three independent experiments. **b** Deacetylation of DosR is dependent on NAD^+^ and is inhibited by NAM. Purified DosR^182Ac^ (8 µM) was incubated with or without Rv1151c (6 µM), NAD^+^ (1 mM), and NAM (10 mM) at 25 °C for 2 h. The acetylation levels were evaluated by western blotting with anti-DosR (1:10,000) at 3-s exposure and anti-K182^Ac^ (1:1000) at 30-s exposure. Results are representative of three independent experiments. **c** The acetylation levels of DosR in H37Ra wild-type (Ra) and *MRA_1161* (*Rv1151c*, homolog in H37Ra)-deletion mutant (KO) in vivo. H37Ra and KO were inoculated into 7H9–10% OADC–0.05% Tween 80 medium and grown to mid-log phase (OD_600_ = 0.4–0.6), respectively. The cultures were incubated with or without NAM (5 mM) for 10 h and collected by centrifugation. Cell extracts (20 μg per lane) were analyzed by western blotting with anti-DosR (1:10,000) at 30-s exposure and anti-K182^Ac^ (1:1000) at 10-min exposure. Equal amounts of loading control for western blotting were analyzed by sodium dodecyl sulfate-polyacrylamide gel electrophoresis and stained with Coomassie blue (Fig. S-a, in Supplementary material). Results are representative of three independent experiments

Lysine acetylation is reversible, and in bacteria, deacetylation is carried out by the sirtuin family of NAD^+^-dependent deacetylases, while nicotinamide (NAM) can inhibit the activity of deacetylases^36^. *NpdA* (*MRA_1161, Rv1151c*) is the only sirtuin homolog found in the *M. tuberculosis* genome. To determine whether DosR was a substrate for NAD^+^-dependent deacetylase, we expressed and purified Rv1151c protein and incubated it at different concentrations with DosR^182Ac^ (high acetylation level) in the presence of NAD^+^. The results showed that DosR K182 could be deacetylated in vitro. Furthermore, DosR^182Ac^ was able to be deacetylated by Rv1151c in a dose-dependent manner, with almost 100% of the acetyl moiety of DosR being removed within 2 h in the presence of 6 µM Rv1151c (Fig. 4a). This result suggested that DosR was a substrate for Rv1151c. We evaluated the inhibitory effect of the small molecule NAM on the deacetylation of DosR. DosR^182Ac^ was not efficiently deacetylated in vitro by Rv1151c in the presence of 10 mM NAM (Fig. 4b).

Next, to determine whether DosR could be deacetylated in vivo in *M. tuberculosis*, we used an *npdA* (*MRA_1161*) knockout strain of H37Ra and compared K182 acetylation levels between the mutant and the wild-type strains. The anti-DosR polyclonal antibody specific for acetylated K182 (anti-K182^Ace^) was used in western blotting and showed that DosR K182 was acetylated in both the wild-type and *npdA* deletion strains, but that the K182 acetylation level in the mutant was significantly increased compared with that in the wild-type strain. The deacetylation effect on K182 DosR in the wild-type strain was inhibited by treatment with 5 mM NAM in the culture medium (Fig. 4c). Taken together, these data suggested that the acetylation of DosR K182 was regulated by NpdA and dependent on NAD^+^, and the deacetylation process could be inhibited by NAM both in vitro and in vivo.

### Acetylation inhibited the transcription of DosR-regulated genes

**Fig. 5:**
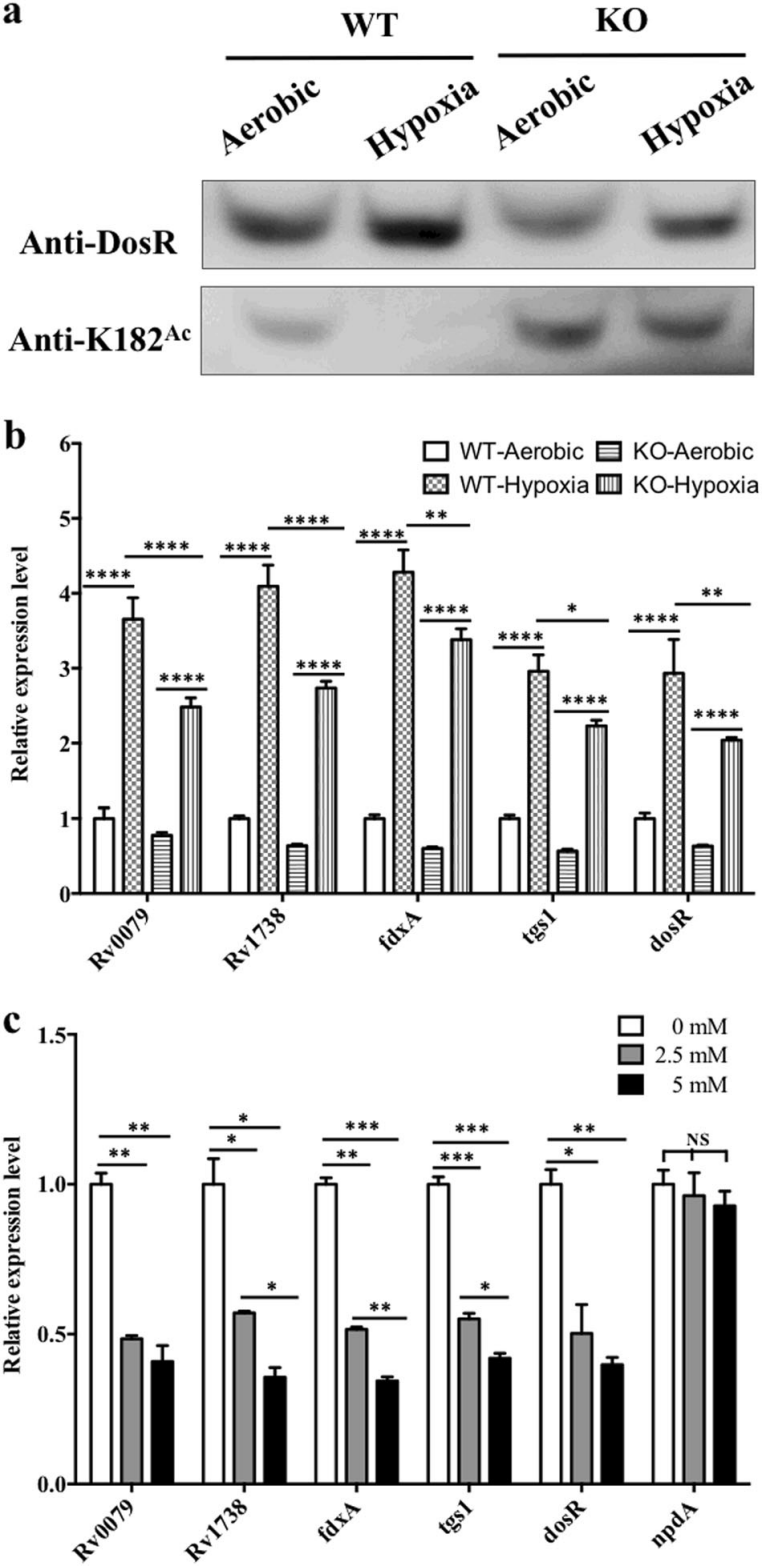
Acetylation of DosR K182 affects gene expression of the DosR regulon in vivo. **a** Acetylation pattern of K182 in DosR from WT and KO mutant under aerobic or hypoxic conditions. The WT and KO mutant bacteria were cultured in Dubos medium at 37 °C for 2 days. The aerobic cultures were grown in 50-ml tubes (10 ml of culture), and hypoxic cultures were grown in 50-ml wax-sealed bottles with a headspace-to-medium ratio of 0.5. Cell extracts (20 μg per lane) were analyzed by western blotting with anti-DosR (1:10,000) at 30-s exposure and anti-K182^Ac^ (1:1000) at 10-min exposure. Equal amounts of loading control for western blotting were analyzed by sodium dodecyl sulfate-polyacrylamide gel electrophoresis and stained with Coomassie blue (Fig. S-b, in Supplementary material). Results are representative of three independent experiments. **b** The transcript levels of candidate genes in the H37Ra strain and KO mutant under aerobic or hypoxic conditions. RNA was isolated from the strains grown in Dubos medium under aerobic or hypoxic conditions (see above), and the transcript levels of the candidate genes were determined by qPCR using the method of 2^−ΔΔCt^. The data were normalized to *rrs* and compared to WT. Two-way ANOVA was performed to compare WT and KO in each condition; *p < 0.05; **p < 0.01; ****p < 0.0001. **c** Analysis of the transcript levels of candidate genes and *npdA* in the H37Ra strain in the presence of NAM. H37Ra was inoculated into 7H9–10% OADC–0.05% Tween 80 medium and grown to mid-log phase (OD_600_ = 0.4–0.6). The cultures were incubated with NAM at different concentrations (0 mM, 2.5 mM, and 5 mM) for 10 h. RNA was isolated, and the transcript levels of candidate genes were analyzed using qPCR. The data were normalized to *rrs*. Statistical analysis was performed by Student’s *t*-test; *p < 0.05; **p < 0.01; ***p < 0.001; NS: not significantly different (*p* > 0.05)

In our previous studies, we observed that an *npdA* deletion mutant exhibited a unique phenotype, such as more growth at low pH and the use of fatty acids as carbon sources, suggesting that acetylation may regulate the ability of *M. tuberculosis* to adapt and persist in host cells^30^. Since the acetylation of K182 abolished DNA binding, we wondered whether K182 DosR of *M. tuberculosis*, when the bacterium was grown under hypoxic conditions, would be deacetylated, thereby interfering with its ability to bind DNA. Deacetylation would result in altered transcript levels of the DosR-regulated genes that are typically affected by bacterial growth in conditions of hypoxia. We hypothesized that the acetylation of K182 when *M. tuberculosis* was grown under aerobic conditions would be higher than that of DosR K182 under hypoxia. Hence, we checked the total protein level and the acetylation level of DosR K182 isolated from the wild-type and *npdA* deletion strains grown under aerobic and hypoxic conditions. For detection of the total protein and acetylated protein, we used the anti-DosR antibody and anti-K182^Ace^ specific antibody, respectively. Western blot analysis revealed that the level of total DosR was significantly higher in wild-type cultures grown under hypoxic conditions than in those grown under aerobic conditions. However, the acetylation level of K182 in the wild-type strain decreased drastically under hypoxia, compared to that under aerobic conditions, to a level below the lower limit of detection (Fig. 5a). This result suggested that *M. tuberculosis* may regulate the DNA-binding ability of DosR by deacetylation of DosR K182 under hypoxic conditions. Moreover, although the total DosR expression in the *npdA* deletion mutant was lower than that in the wild-type strain grown under both aerobic and hypoxic conditions, the acetylation level of the *npdA* deletion mutant was higher. This result again confirmed that the acetylation of DosR K182 was regulated by NpdA in *M. tuberculosis*.

As a major response regulator, DosR induces the expression of approximately 48 genes in the genome, including itself^8,16^. To determine whether acetylation affected the activity of DosR as a transcriptional regulator, several DosR-regulated genes including *Rv0079*, *Rv1738*, *fdxA*, *tgs1*, and *dosR* itself were selected to be evaluated. The regulation of DosR activity under hypoxia in both the *npdA* deletion-mutant and wild-type strains was investigated. Quantitative real-time PCR (qPCR) results showed that the DosR-regulated genes, including *dosR* itself, were all significantly downregulated in the mutant strain compared with those in the wild-type strain (Fig. 5b).

Since the deacetylation of K182 was regulated by NpdA (Figs. 4a, b), and NAM inhibited this process, resulting in an increase in K182 acetylation in vivo (Fig. 4c), we determined whether NAM affected the transcription of the DosR-regulated genes in *M. tuberculosis*. Total RNA was isolated from the bacteria, and the transcript levels were determined by qPCR. NAM caused reduced transcription of DosR-regulated genes in a dose-dependent manner. The addition of NAM to a concentration of 2.5 mM in H37Ra culture media resulted in the downregulation of transcript levels for all the candidate genes evaluated in this study, to approximately 50% of the levels seen when *M. tuberculosis* was grown in the absence of NAM (Fig. 5c). At the same time, the transcription of *npdA* was not significantly affected by NAM at any of the concentrations tested (Fig. 5c). Taken together, these results confirmed that acetylation of K182 regulated the DNA-binding ability of DosR and the transcript levels of DosR-regulated genes in *M. tuberculosis* when grown under hypoxic conditions.

## Discussion

The success of *M. tuberculosis* as a pathogen is partly owing to its ability to adapt to and persist in a limited-oxygen microenvironment. DosR regulon function is believed to be essential for bacterial survival during latency. Mass spectrometry results from our group and others pertaining to the lysine acetylome indicate that K182 of DosR is capable of being acetylated^30,31^. We hypothesized that lysine acetylation of DosR may play a critical role in *M. tuberculosis* adaptation to the hypoxic microenvironment within the host.

In the current study, our results from EMSA showed that the K182Q DosR mutant, unlike the K182R mutant, lost its ability to bind DNA. Positive-charge neutralization in the K-to-Q mutant is believed to mimic the acetylation of DosR and to behave similarly to acetylation. In addition, DosR^182Ac^, with specific acetylation of the lysine 182 residue achieved through genetic methods, displayed a loss in DNA-binding ability as determined by EMSA and ITC assays. Therefore, we consider that acetylation at K182 played a key role in the DNA-binding activity of DosR. It is known that the positively charged lysine residue has an affinity for negatively charged DNA because of charge forces. After being acetylated, the K182 residue no longer carried a positive charge, and consequently, acetylation caused the DosR-DNA binding force to be attenuated. Moreover, K182 is located in the middle of helix 9, and its side chain forms hydrogen bonds with G13 and A12, which play the most important role in target-sequence recognition^19,20,32^. According to structure-based modeling, DosR182K and the K182R mutant could form two H-bonds with the target DNA. The shorter side chain of glutamine (for K182Q) and the acetylated lysine (for K182Ac) caused exceedingly distant or close contact between the residue-182 side chain and the DNA bases, which weakened the hydrogen bonds or pushed the target DNA away. Therefore, the molecular mechanism of the observed loss in DNA-binding ability that resulted from the acetylation of K182 was probably due to neutralization of the positive charge on K182 and a change in distance between the K182 side chain and the target DNA. Both these changes affected the formation of H-bonds with the DNA and ultimately nullified the ability of DosR to bind DNA.

**Fig. 6:**
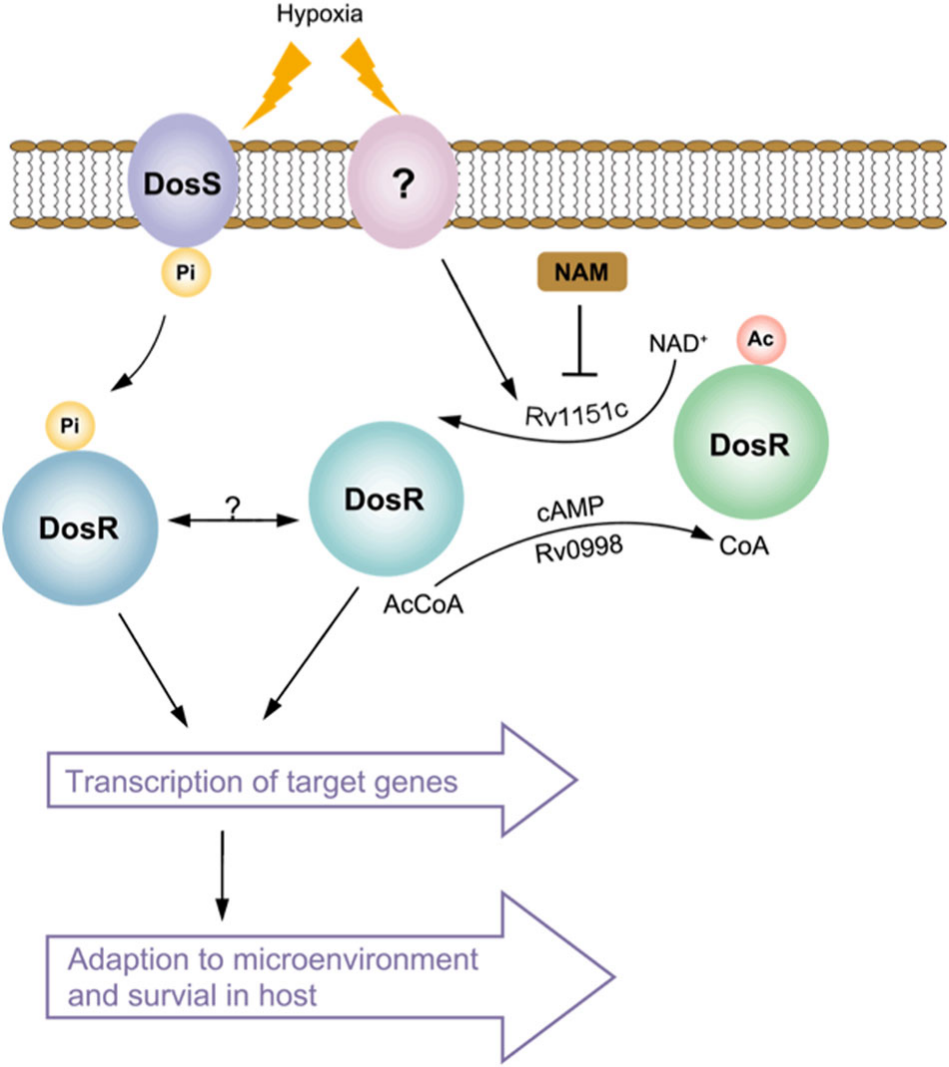
Acetylation is involved in regulating DosR activity in *M. tuberculosis*. Active DosR condition: deacetylated DosR by Rv1151c enhances DNA-binding ability of DosR and then promotes the transcriptional activity of target genes, allowing *M. tuberculosis* to adapt to hypoxia and transition to latency in host. Inactive DosR condition: acetylated DosR by Rv0998 inhibits DNA-binding ability, which abolishes transcriptional activity of target genes. Ac acetylation, Pi phosphorylation, Ac-CoA acetyl-coenzyme A, NAD+ nicotinamide adenine dinucleotide, NAM nicotinamide

Our results showed that DosR could be deacetylated by NAD-dependent deacetylase Rv1151c (NpdA) in vitro, and DosR K182 was hyperacetylated in the *npdA* deletion-mutant strain, indicating that NpdA also regulates deacetylation of DosR K182 in vivo. We further observed that the transcript level of DosR-regulated genes, including *dosR*, was downregulated in the *npdA* deletion strain, compared with that in the wild-type strain. These results demonstrated that the acetylation level of K182 in the DNA-binding domain affects the function of DosR as a transcriptional regulator. Another noteworthy observation was that acetylation of DosR K182 in *M. tuberculosis* was significantly different between bacteria grown under hypoxia and those grown under aerobic conditions, suggesting that hypoxia induces deacetylation of DosR K182 in *M. tuberculosis*. Given that the transcription of DosR-regulated genes was not lost completely in the *npdA* deletion strain, we propose that acetylation and deacetylation of DosR may together regulate the biological functions of DosR. Interestingly, one recent study showed that acetyltransferase Rv0998 could acetylate DosR K182 and proposed that hypoxia may induce the deacetylation of DosR^37^. However, the deacetylation mechanism of DosR was not determined in that study. Combining these results with those of our study, it has been clearly shown that DosR deacetylation by Rv1151c causes an active DosR condition and enhances the DNA-binding ability of DosR to promote transcriptional activity in *M. tuberculosis* under hypoxia and that DosR acetylation by Rv0998 causes an inactive DosR condition and inhibits its DNA-binding ability, abolishing transcriptional activity (Fig. 6). These findings suggest that the reversible acetylation of DosR K182 is critical in regulating DosR activity and the virulence of *M. tuberculosis*. Several published studies have suggested that protein acetylation plays a critical role in bacterial virulence, which indicates a new modulation mechanism for bacterial pathogenesis^38^. However, more studies are required to unveil the underlying mechanism of protein acetylation as it relates to bacterial virulence modulation.

It has been shown that phosphorylation of DosR is required for the activation of transcription of DosR-regulated genes and that the D54 residue is involved in the phosphorylation of DosR^14,18,21^. However, the fact that unphosphorylated DosR and a D54E mutant protein are able to interact with target DNA provides a confusing result^14,21,39^. Our study demonstrated that phosphorylation increased the DNA affinity of DosR and K182R in vitro (data not shown). Based on reduced DNA binding as a result of acetylation of K182 and its low level of acetylation under hypoxia, we hypothesized that the activity of DosR may be regulated not only by phosphorylation but also by lysine acetylation. Since DosR regulates approximately 48 different genes that are involved in *M. tuberculosis* latency in host cells, DosR regulation with only one phosphorylation-dependent “on–off” switch may not guarantee adequate regulatory accuracy of gene expression and proper response to a multitude of stresses in diverse host microenvironments. In fact, there are other reports supporting DNA-binding activity being regulated by acetylation and phosphorylation, as observed for the transcriptional regulator RcsB in *E. coli*, controlling cell division and flagellum biosynthesis^25^, and PhoP, a member of an important two-component system regulating *Salmonella* virulence^24^. Since hypoxia induces deacetylation of DosR K182 in *M. tuberculosis*, we propose K182 acetylation in DosR as another “on–off” switch. When *M. tuberculosis* encounters a microenvironmental signal such as hypoxia in the host, the activation of DosR may be regulated, not only by DosS/DosT sensing the environmental signal and the phosphotransfer to DosR but also by reversible acetylation of DosR itself at K182. It is not clear whether there is crosstalk between phosphorylation of the D54 residue and acetylation of the K182 residue of DosR. The possibility of such mutual promotion or blocking of these two types regulation of DosR requires further investigation.

In this study, we showed that unacetylated K182 was representative of activated DosR and was beneficial for the transcription of DosR regulon genes in *M. tuberculosis* during exposure to hypoxia. Moreover, NAM, an inhibitor of deacetylase, caused the level of transcription of DosR-regulated genes to be reduced (Fig. 5c). Targeting the deacetylation of K182 in DosR may be a novel strategy for blocking the DosR signaling that is essential for *M. tuberculosis* adaptation and survival in host cells. In fact, NAM has demonstrated activity against nonreplicating, persistent *M. tuberculosis* infections in vitro^40^ and in vivo^41,42^, and pyrazinamide (PZA), an important first-line drug during shortages of TB treatment, is synthesized as an analog of NAM. However, NAM, as an intermediate metabolite and a general deacetylase inhibitor in most species, may exhibit toxicity to host cells. Although NAM is not an ideal drug candidate itself, our findings suggest that targeting this novel reversible regulation mechanism of key proteins is a viable strategy in anti-TB drug development, as inhibitors of histone deacetylase have been approved for cancer treatment^43^.

## Materials and methods

### Bacterial strains

NpdA is the only NAD^+^-dependent deacetylase found in the *M. tuberculosis* genome^44^. NpdA is encoded by *Rv1151c* in strain H37Rv and *MRA_1161* in H37Ra. H37Ra and its *npdA* deletion mutant (∆*npdA*) were used in this study. The *ΔnpdA* strain was constructed using the specialized transducing phage system^30^. *E. coli* DH5α was used for genetic manipulation of the DNA, and *E. coli* Rosetta was used for expression of the recombinant DosR proteins.

### Expression and purification of DosR and site-directed mutants

The *dosR* DNA was first amplified using primers DM1 and DM2 and cloned into pET28a using restriction enzymes *NdeI* and *HindIII* (New England Biolabs, Beijing, China) to generate the recombinant plasmid pET28a-*dosR*, which was verified by sequencing.

The pET28a-*dosR* plasmid was used as the template for site-directed mutagenesis, which was carried out using the KOD Mut Kit (Toyobo, Toyo, Japan). The primers K182R1 and K182R2 were used for site-directed mutagenesis to replace AAG (K182) with CGA (R182) for pET28a-*dosR*^182R^, and the primers K182Q1 and K182Q2 were used to change AAG (K182) to CAG (Q182) for pET28a-*dosR*^182Q^. These point mutations were confirmed by DNA sequencing. The constructed plasmids were individually transformed into *E. coli* strain Rosetta cells, and the expression of DosR proteins was induced with 1 mM isopropyl-β-D-1-thiogalactopyranoside (IPTG, Sinopharm, Shanghai, China) at 30 °C for 4 h. The collected bacteria were lysed and then centrifuged at 13,000 rpm for 30 min to clarify the supernatant. The proteins were purified by Ni^2+^-affinity chromatography (Qiagen, Shanghai, China) following the manufacturer’s instructions. Sequences for all the primers used in this study are shown in Table 1.

**Table 1:** Primers used in this study

Primer name	Sequence
DM1	5′ GGGCATATGGTGGTAAAGGTCTTCTTGGT 3′
DM2	5′ TTAAAGCTTTCATGGTCCATCACCGGGTG 3′
K182R1	5′ AAAGACGGTGCGAAACTACGTGTCGCGGTTG 3′
K182R2	5′ ACACGTAGTTTCGCACCGTCTTTTCGGCTAGG 3′
K182Q1	5′ AAAGACGGTGCAGAACTACGTGTCGCGGTTGCT 3′
K182Q2	5′ ACACGTAGTTCTGCACCGTCTTTTCGGCTAGGAA 3′
EM1	5′ TTGGGGACTAAAGTCCCTAA 3′
EM2	5′ TTAGGGACTTTAGTCCCCAA 3′
PTD1	5′ TGGCATATGGTGGTAAAGGTCTTCTTGGT 3′
PTD2	5′ TTGAAGCTTTCAATGATGATGATGATGATGTGGTCCATCAC CGGGTGGCC 3′
PTD3	5′ GGGCATATGGTGCAGGACCCGCTATCAG 3′
K182Ac1	5′ AAAGACGGTGTAGAACTACGTGTCGCGGTTG 3′
K182Ac2	5′ ACACGTAGTTCTACACCGTCTTTTCGGCTAGG 3′
PR1	5′ AAACATATGATGCGAGTGGCGGTGCTCAG 3′
PR2	5′ AAAAAGCTTCTATTTCAGCAGGGCGGGCAG 3′
Rv0079 F	5′ TGCCGCTGATTGTGAATT 3′
Rv0079 R	5′ AACAGGAATGGCAGTCCA 3′
Rv1738 F	5′ AGGAATTGGTGGGTGTTG 3′
Rv1738 R	5′ CCTTCAACATTCGCTTCC 3′
Rv2007c F	5′ CTATGTGATCGGTAGTGAGT 3′
Rv2007c R	5′ GGTTGATGTAGAGCATTCG 3′
Rv3130c F	5′ AAGGCAGAAGACGTGGAT 3′
Rv3130c R	5′ CGAGCGACGATAAGAAGG 3′
Rv3133c F	5′ CAACGGCATTGAACTGTG 3′
Rv3133c R	5′ CTCGTCAGAGGTGTAGGA 3′
Rv1151c F	5′ GTTGTGGTGTGCCCTACA 3′
Rv1151c R	5′ GCTCACCGAACCATACGA 3′
16S(rrs) F	5′ GCGAGGTTAAGCGAATCC 3′
16S(rrs) R	5′ TAGCGACTCCGACTTCAC 3′

### Generation of homogenous DosR protein containing Nɛ-acetyllysine at K182

To generate a recombinant DosR protein that homogeneously contained acetylated K182, we used a genetic engineering system to direct the site-specific incorporation of N^ɛ^-acetyllysine to the amber codon^35^. We cloned wild-type *dosR* or the gene encoding the C-terminal domain of DosR (amino acid residues 144–217) into pT7 to form pT7-*dosR* or pT7-*dosR*-C using primers PTD1 and PTD2 or PTD3 and PTD2, and replaced the codon for K182 (AAG) with an amber codon (TAG) using site-directed mutagenesis PCR with primers K182Ac1 and K182Ac2. The site-specific mutated recombinant plasmids were verified by sequencing.

The resulting plasmids, and two additional plasmids (pAcKRS-3 and pPylT)^35^, were transformed into in *E. coli* Rosetta cells and plated on agar plates containing spectinomycin (50 μg/ml), kanamycin (50 μg/ml), and ampicillin (150 μg/ml). Recombinant strains of *E. coli* containing the three plasmids were grown in LB media supplemented with the same antibiotics to mid-log phase (OD_600_ = 0.6). The culture was then supplemented with 1 mM IPTG, 2 mM N-acetyllysine (Sigma-Aldrich, Santa Clara, CA, USA), and 10 mM NAM (Sigma-Aldrich) and incubated at 25 °C for 18 h (for wild-type DosR^182Ac^) or 30 °C for 14 h (for DosR-C^182Ac^). The DosR and DosR-C proteins containing acetylated K182 were purified by Ni^2+^-affinity chromatography (Qiagen) following the manufacturer’s instructions.

### Electrophoretic mobility shift assay (EMSA)

EMSA was used to test the binding of DNA probes with DosR as previously reported^14^, using oligonucleotides EM1 and EM2 as probes^20^. Briefly, the two probes were heated to 95 °C and annealed by gradually cooling to room temperature (approximately 25 °C) to form double-stranded oligonucleotides. The binding of DosR and the double-stranded oligonucleotides was carried out by incubation at 37 °C for 30 min in 15 μl of reaction mixture containing 2 μM DNA; 24 mM Tris-HCl buffer, pH 7.5; and 20 mM MgCl_2_. Following the incubation, the entire reaction volume was electrophoresed on a 12% nondenaturing Tris-borate EDTA polyacrylamide gel (12% acrylamide-bisacrylamide (29:1), 89 mM Tris, 89 mM borate, 2 mM EDTA, pH 8.0, Bio-Rad, Hercules, CA, USA). The gel was stained with ethidium bromide and visualized with a transilluminator at 312 nm. The assays were repeated three times.

### Preparation of antisera

The antibodies were customized by GL Biochem (Shanghai) Ltd. To generate antibodies, the peptide KLH (CEKTV(K-Ac) NYVSRLLAK) was synthesized, and DosR protein was expressed and purified from *E. coli* BL21 cells harboring the pET28a-*dosR* plasmid as described above, which were used to immunize rabbits for the production of a polyclonal antibody. The study was approved by the ethics committee at GL Biochem (Shanghai) Ltd. (No. 20). Rabbits were repeatedly immunized twice using 350 μg protein or peptide with Freund’s adjuvant (complete) and 150 μg protein with Freund’s adjuvant (incomplete) by six subcutaneous injections during the 10-week period, and 30–45 ml of the antiserum from each rabbit was collected. The control peptide CEKTVKNYVSRLLAK was used to remove nonspecific antibody from the anti-K182_Ace_ antisera.

### Western blot analysis

Standard western blot procedures were used in this study. Purified recombinant protein (0.2 μg) or cell extracts (50 μg) were separated by 12% sodium dodecyl sulfate-polyacrylamide gel electrophoresis and transferred to a polyvinylidene difluoride membrane. The primary antibodies used in the corresponding blot were anti-DosR (1:10,000) or anti-K182^Ac^ (1:1000). After incubation with primary antibodies, the membranes were washed and incubated with horseradish peroxidase-conjugated antirabbit secondary antibodies, followed by detection using enhanced chemiluminescence western blot substrate (Bio-Rad).

### Expression and purification of Rv1151c

The *Rv1151c* gene was first cloned into pET28a using primers PR1 and PR2 to form pET28a-*Rv1151c*. The constructed plasmid was transformed into *E. coli* strain Rosetta cells, and expression of the Rv1151c protein was induced with 1 mM IPTG for 15 h at 25 °C or once an OD_600_ of 0.6 was obtained. The proteins were purified using Ni^2+^-affinity chromatography (Qiagen) following the manufacturer’s instructions.

### Isothermal titration calorimetry (ITC)

The dissociation constant (*Kd*) of the interaction between DNA and DosR or DosR-C was measured by ITC using a MicroCal iTC200 calorimeter (GE Healthcare, Chicago, IL, USA). Calorimetric titration of DosR or DosR^182Ac^ (700 μM) to DNA (20 μM) and titration of DosR-C or DosR-C^182Ac^ (350 μM) to DNA (10 μM) were performed at 25 °C in an assay buffer containing 20 mM Tris (pH 8.0) and 200 mM NaCl. The time between injections was 150 s. The ITC data were analyzed by integrating the heat effects after the data were normalized to the amount of injected protein. Data fitting was conducted to determine the dissociation constant based on a single-site binding model using the Origin software package (MicroCal).

### Deacetylase activity

Deacetylation of DosR by the *M. tuberculosis* deacetylase Rv1151c was performed at 25 °C for 2 h in deacetylation reaction buffer containing 50 mM Tris-HCl (pH 8.0), 135 mM NaCl, 2.5 mM KCl, and 1 mM MgCl_2_ in the presence or absence of 1 mM NAD^+^ and in the presence or absence of 5 mM or 10 mM NAM. Specific protein and NAM concentrations are indicated in the figure legends.

### Quantitative real-time PCR

*M. tuberculosis* H37Ra and the MRA_1161 deletion-mutant strain (∆*npdA*) were inoculated into 7H9–OADC–0.05% Tween 80 medium (Becton Dickinson, Sparks, MD, USA) and cultured at 37 °C for 7 days until the log phase (OD_600_ 0.4–0.6) was reached. The cells were collected by centrifugation and then washed twice with 20 mM phosphate-buffered saline (pH 6.8). The bacteria were then resuspended in Dubos Tween albumin medium (Becton Dickinson). The aerobic cultures were simultaneously grown in 50-ml culture tubes (10 ml of culture) placed in a shaker incubator at 37 °C for 2 days at 100 rpm. The hypoxic cultures were incubated in Dubos Tween albumin medium in 50-ml bottles with a headspace-to-medium ratio of 0.5, sealed with wax, and cultured at 37 °C for 2 days. The cultures of ∆*npdA* described above contained 75 µg/ml hygromycin. The cultures of strain H37Ra grown to mid-log phase in 7H9-OADC medium were supplemented with NAM at different concentrations (0 mM, 2.5 mM, and 5 mM) and incubated at 37 °C for 10 h. All cultures were pelleted, and the pellets were snap-frozen in liquid nitrogen and then stored at −80 °C. The stored pellets were processed by disruption using bead beaters, and the total RNA was extracted using TRIzol reagent (Invitrogen, Shanghai, China) followed by DNase I digestion, according to a previously described protocol^45^. The cDNA was synthesized from 1 μg of the total RNA using a reverse transcription kit (Takara, Beijing, China). qPCR was performed with the Roche LightCycler 96 System using SYBR Green with 16S (*rrs*) as an internal control. Primers for the qPCR are listed in Table 1. The relative transcript levels for each reference gene were calculated by the method of 2^−ΔΔCt^.

## Electronic supplementary material

Supplementary information

## References

[1] The World Health Organization. *Global tuberculosis report 2016* (2016).

[2] North RJ, Jung YJ (2004). Immunity to tuberculosis. Annu. Rev. Immunol..

[3] Stewart GR, Robertson BD, Young DB (2003). Tuberculosis: a problem with persistence. Nat. Rev. Microbiol..

[4] Chao MC, Rubin EJ (2010). Letting sleeping dos lie: does dormancy play a role in tuberculosis?. Annu. Rev. Microbiol.

[5] Wayne LG, Sohaskey CD (2001). Nonreplicating persistence of Mycobacterium tuberculosis. Annu. Rev. Microbiol.

[6] Boon C, Dick T (2002). Mycobacterium bovis BCG response regulator essential for hypoxic dormancy. J. Bacteriol..

[7] Dasgupta N (2000). Characterization of a two-component system, devR-devS, of Mycobacterium tuberculosis. Tuber. Lung Dis..

[8] Park HD (2003). Rv3133c/dosR is a transcription factor that mediates the hypoxic response of Mycobacterium tuberculosis. Mol. Microbiol..

[9] Bretl DJ, Demetriadou C, Zahrt TC (2011). Adaptation to environmental stimuli within the host: two-component signal transduction systems of Mycobacterium tuberculosis. Microbiol. Mol. Biol. Rev..

[10] Kendall S (2004). The Mycobacterium tuberculosis dosRS two-component system is induced by multiple stresses. Tuberculosis.

[11] Voskuil MI (2003). Inhibition of respiration by nitric oxide induces a Mycobacterium tuberculosis dormancy program. J. Exp. Med..

[12] Kumar A, Toledo JC, Patel RP, Lancaster JR, Steyn AJ (2007). Mycobacterium tuberculosis DosS is a redox sensor and DosT is a hypoxia sensor. Proc. Natl Acad. Sci. USA.

[13] Honaker RW, Leistikow RL, Bartek IL, Voskuil MI (2009). Unique roles of DosT and DosS in DosR regulon induction and Mycobacterium tuberculosis dormancy. Infect. Immun..

[14] Roberts DM, Liao RP, Wisedchaisri G, Hol WG, Sherman DR (2004). Two sensor kinases contribute to the hypoxic response of Mycobacterium tuberculosis. J. Biol. Chem..

[15] Sherman DR (2001). Regulation of the Mycobacterium tuberculosis hypoxic response gene encoding α-crystallin. Proc. Natl Acad. Sci. USA.

[16] Gautam US, Sikri K, Vashist A, Singh V, Tyagi JS (2014). Essentiality of DevR/DosR interaction with SigA for the dormancy survival program in Mycobacterium tuberculosis. J. Bacteriol..

[17] Sivaramakrishnan S, Ortiz de Montellano PR (2013). The DosS-DosT/DosR mycobacterial sensor system. Biosensors.

[18] Saini DK (2004). DevR–DevS is a bona fide two-component system of Mycobacterium tuberculosis that is hypoxia-responsive in the absence of the DNA-binding domain of DevR. Microbiology.

[19] Wisedchaisri G, Wu M, Sherman DR, Hol WG (2008). Crystal structures of the response regulator DosR from Mycobacterium tuberculosis suggest a helix rearrangement mechanism for phosphorylation activation. J. Mol. Biol..

[20] Wisedchaisri G (2005). Structures of Mycobacterium tuberculosis DosR and DosR–DNA complex involved in gene activation during adaptation to hypoxic latency. J. Mol. Biol..

[21] Chauhan S, Tyagi JS (2008). Cooperative binding of phosphorylated DevR to upstream sites is necessary and sufficient for activation of the Rv3134c-devRS operon in Mycobacterium tuberculosis: implication in the induction of DevR target genes. J. Bacteriol..

[22] Minch K, Rustad T, Sherman DR (2012). Mycobacterium tuberculosis growth following aerobic expression of the DosR regulon. PLoS ONE.

[23] Flores-Valdez MA (2015). Overexpression of DosR in Mycobacterium tuberculosis does not affect aerobic replication in vitro or in murine macrophages. Ann. Microbiol.

[24] Ren J (2016). Acetylation of lysine 201 inhibits the DNA-binding ability of PhoP to regulate Salmonella virulence. PLoS Pathog..

[25] Hu LI (2013). Acetylation of the response regulator RcsB controls transcription from a small RNA promoter. J. Bacteriol..

[26] Li R (2010). CobB regulates Escherichia coli chemotaxis by deacetylating the response regulator CheY. Mol. Microbiol..

[27] Sang Y (2016). Protein acetylation is involved in Salmonella enterica serovar Typhimurium virulence. J. Infect. Dis..

[28] Sang Y (2017). Acetylation regulating protein stability and DNA-binding ability of HilD, thus modulating Salmonella typhimurium virulence. J. Infect. Dis..

[29] Castaño‐Cerezo S (2014). Protein acetylation affects acetate metabolism, motility and acid stress response in Escherichia coli. Mol. Syst. Biol..

[30] Bi J (2017). Modulation of central carbon metabolism by acetylation of isocitrate lyase in Mycobacterium tuberculosis. Sci. Rep..

[31] Liu F (2014). Acetylome analysis reveals diverse functions of lysine acetylation in Mycobacterium tuberculosis. Mol. Cell. Proteomics.

[32] Gupta RK, Chauhan S, Tyagi JS (2011). K182G substitution in DevR or C8G mutation in the Dev box impairs protein–DNA interaction and abrogates DevR‐mediated gene induction in Mycobacterium tuberculosis. FEBS J..

[33] Gupta RK, Thakur TS, Desiraju GR, Tyagi JS (2009). Structure-based design of DevR inhibitor active against nonreplicating Mycobacterium tuberculosis. J. Med. Chem..

[34] Hu LI, Lima BP, Wolfe AJ (2010). Bacterial protein acetylation: the dawning of a new age. Mol. Microbiol..

[35] Neumann H, Peak-Chew SY, Chin JW (2008). Genetically encoding Nε-acetyllysine in recombinant proteins. Nat. Chem. Biol..

[36] Gu J (2009). Cloning and characterization of NAD-dependent protein deacetylase (Rv1151c) from Mycobacterium tuberculosis. Biochemistry (Mosc.).

[37] Yang H (2018). Lysine acetylation of DosR regulates the hypoxia response of Mycobacterium tuberculosis. Emerg. Microbes Infect..

[38] Ren J, Sang Y, Lu J, Yao YF (2017). Protein acetylation and its role in bacterial virulence. Trends Microbiol..

[39] Bagchi G, Chauhan S, Sharma D, Tyagi JS (2005). Transcription and autoregulation of the Rv3134c-devR-devS operon of Mycobacterium tuberculosis. Microbiology.

[40] McDermott W, Tompsett R (1954). Activation of pyrazinamide and nicotinamide in acidic environments in vitro. Am. Rev. Tuberc..

[41] Mackaness G (1956). The intracellular activation of pyrazinamide and nicotinamide. Am. Rev. Tuberc..

[42] Murray MF (2003). Nicotinamide: an oral antimicrobial agent with activity against both Mycobacterium tuberculosis and human immunodeficiency virus. Clin. Infect. Dis..

[43] Marks PA, Richon VM, Miller T, Kelly WK (2004). Histone deacetylase inhibitors. Adv. Cancer Res..

[44] Cole S (1998). Deciphering the biology of Mycobacterium tuberculosis from the complete genome sequence. Nature.

[45] Benjak, A., Sala, C. & Hartkoorn, R. C. in *Mycobacteria Protocols* (eds Parish, T. & Roberts, D. M.) (Ch. 2) (Humana Press Inc., New York, NY, USA, 2015)

