# Supplementary material for: Acetylation of lysine 182 inhibits the ability of Mycobacterium tuberculosis DosR to bind DNA and regulate gene expression during hypoxia
Source: Emerg Microbes Infect. 2018 Jun 13;7:108. doi: 10.1038/s41426-018-0112-3 (PMC5999986; doi:10.1038/s41426-018-0112-3)
Supplement: Supplementary file 1 — Supplementary information [file 41426_2018_112_MOESM1_ESM.docx]

**Acetylation of Lysine 182 Inhibits the DNA-binding Ability of *Mycobacterium tuberculosis* DosR to Regulate Gene Expression during Hypoxia**

**Running Title:** Lysine 182 Acetylation Inhibits DNA-binding of Mtb DosR

**Author Names:** Jing Bi^1#^, Zongchao Gou^1#^, Fengzhu Zhou^1^, Yiqing Chen^1^, Jianhua Gan^1^, Jun Liu^1,2^, Honghai Wang^1^, Xuelian Zhang^1*^

**Author Affiliations:**

^1^State Key Laboratory of Genetic Engineering, School of Life Science, Fudan University, Shanghai, China, 200438.  ^2^Department of Molecular Genetics, Faculty of Medicine, University of Toronto, Toronto, Ontario, Canada, M5G 1M1.

**^#^** **These authors** contributed equally to this work.

*** Corresponding author** : Xuelian Zhang ([xuelianzhang@fudan.edu.cn](mailto:xuelianzhang@fudan.edu.cn)).

State Key Laboratory of Genetic Engineering, School of Life Science, Fudan University, Shanghai, China, 200438.

Telephone number: 0086-21-51630587

Fax number: 0086-21-51630587

**
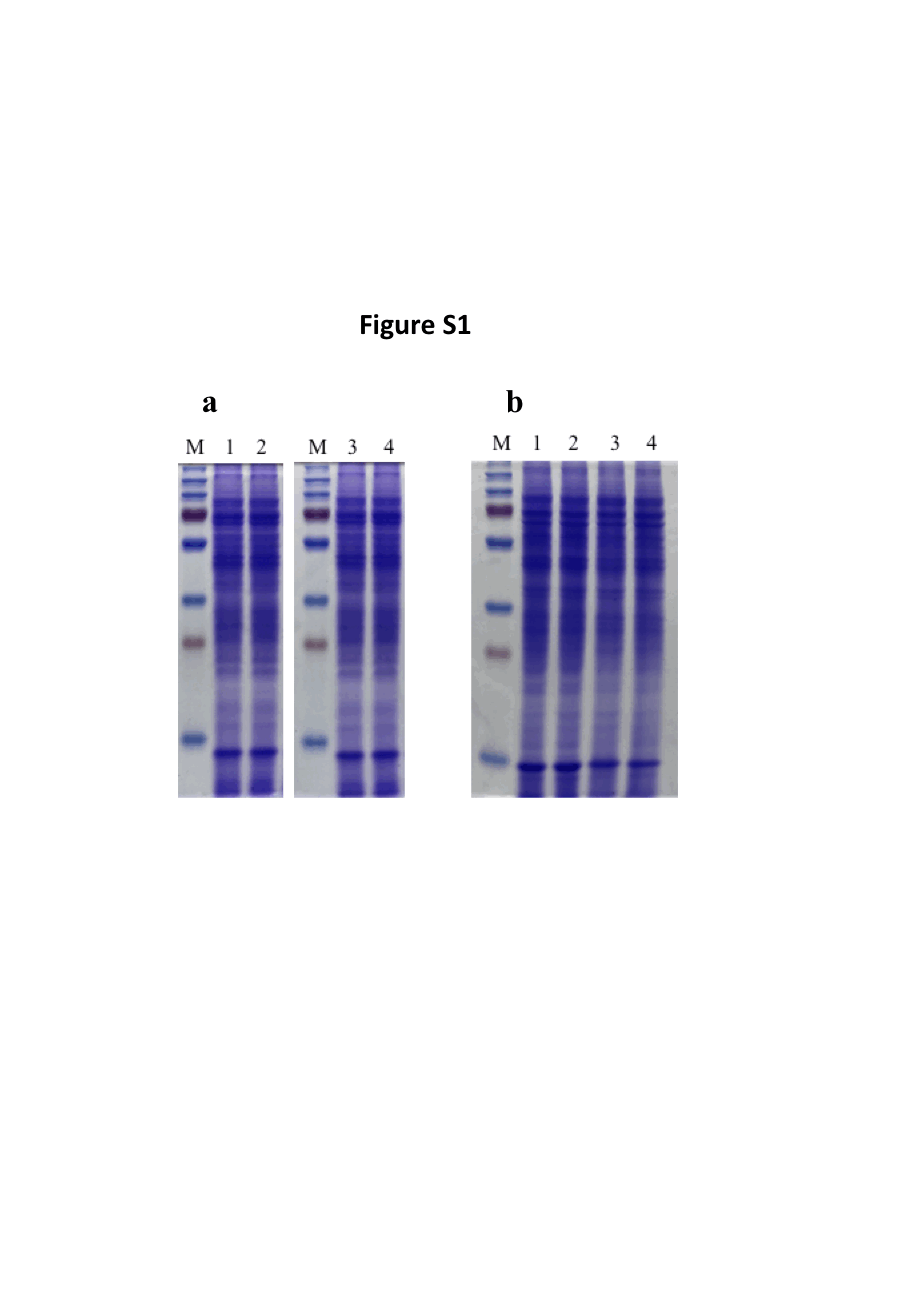
**

**Fig. S1. Loading control for Western blotting analysis. (A) loading control for Fig. 4c.**The H37Ra wild-type (Ra) and *MRA_1161* (*Rv1151c,* homolog in H37Ra)-deletion mutant (KO) were inoculated into 7H9-10% OADC-0.05% Tween 80 medium to mid-log phase (OD_600_ = 0.4–0.6), respectively. The cultures were added with NAM (5mM) or without, and incubated for 10 h, and collected by centrifugation. Cell extracts (20 μg per lane) was analyzed by SDS-PAGE and stained with Coomassie blue. M: marker; lane 1: Ra (without NAM); lane 2: KO (without NAM); lane 3: Ra (with 5mM NAM); lane 4: KO (with 5mM NAM). (**B**) **Loading control for Fig. 5a.** The wild-type (WT) and KO mutant bacteria were cultured in Dubos medium under aerobic or hypoxic conditions and cell extracts were prepared and equal amount (20 μg/lane) were analyzed by SDS-PAGE and stained with Coomassie blue. M: marker; lane 1: Ra (under aerobic conditions); lane 2: Ra (under hypoxic conditions); lane 3: KO (under aerobic conditions); lane 4: KO (under hypoxic conditions).
